# Autoantibody Profiling on Human Proteome Microarray for Biomarker Discovery in Cerebrospinal Fluid and Sera of Neuropsychiatric Lupus

**DOI:** 10.1371/journal.pone.0126643

**Published:** 2015-05-08

**Authors:** Chaojun Hu, Wei Huang, Hua Chen, Guang Song, Ping Li, Qiang Shan, Xuan Zhang, Fengchun Zhang, Heng Zhu, Lin Wu, Yongzhe Li

**Affiliations:** 1 Department of Rheumatology and Clinical Immunology, Peking Union Medical College Hospital, Chinese Academy of Medical Sciences & Peking Union Medical College, Key Laboratory of Rheumatology and Clinical Immunology, Ministry of Education, Beijing, China; 2 CAS Key Laboratory of Genome Sciences and Information, Beijing Institute of Genomics, Chinese Academy of Sciences, Beijing, China; 3 Department of Pharmacology and Molecular Sciences, Johns Hopkins University School of Medicine, Baltimore, Maryland, United States of America; Nippon Medical School Graduate School of Medicine, JAPAN

## Abstract

Autoantibodies in cerebrospinal fluid (CSF) from patients with neuropsychiatric systemic lupus erythematosus (NPSLE) may be potential biomarkers for prediction, diagnosis, or prognosis of NPSLE. We used a human proteome microarray with~17,000 unique full-length human proteins to investigate autoantibodies associated with NPSLE. Twenty-nine CSF specimens from 12 NPSLE, 7 non-NPSLE, and 10 control (non-systemic lupus erythematosus)patients were screened for NPSLE-associated autoantibodies with proteome microarrays. A focused autoantigen microarray of candidate NPSLE autoantigens was applied to profile a larger cohort of CSF with patient-matched sera. We identified 137 autoantigens associated with NPSLE. Ingenuity Pathway Analysis revealed that these autoantigens were enriched for functions involved in neurological diseases (score = 43).Anti-proliferating cell nuclear antigen (PCNA) was found in the CSF of NPSLE and non-NPSLE patients. The positive rates of 4 autoantibodies in CSF specimens were significantly different between the SLE (i.e., NPSLE and non-NPSLE) and control groups: anti-ribosomal protein RPLP0, anti-RPLP1, anti-RPLP2, and anti-TROVE2 (also known as anti-Ro/SS-A). The positive rate for anti-SS-A associated with NPSLE was higher than that for non-NPSLE (31.11% cf. 10.71%; *P* = 0.045).Further analysis showed that anti-SS-A in CSF specimens was related to neuropsychiatric syndromes of the central nervous system in SLE (*P* = 0.009). Analysis with Spearman’s rank correlation coefficient indicated that the titers of anti-RPLP2 and anti-SS-A in paired CSF and serum specimens significantly correlated. Human proteome microarrays offer a powerful platform to discover novel autoantibodies in CSF samples. Anti-SS-A autoantibodies may be potential CSF markers for NPSLE.

## Introduction

Systemic lupus erythematosus (SLE) is a systemic autoimmune disease characterized by production of pathogenic autoantibodies and multiple organ and tissue damage[1]. Involvement of the nervous system, or neuropsychiatric systemic lupus erythematosus (NPSLE), is an important subtype of SLE that encompass a wide range of manifestations, including aseptic meningitis, psychosis and seizures, but lacks optimized diagnostic approaches[2]. The clinical diagnosis of NPSLE remains challenging. Therefore, novel biomarkers of NPSLE are urgently needed for clinical practice.

Cerebrospinal fluid (CSF)contains metabolic products of the central nervous system, and pathology in the central nervous system can be reflected in the traits and composition of the CSF. For example, a variety of autoantibodies have been detected in the CSF of SLE patients, including anti-glutamate receptor ɛ2 subunit (GluRɛ2)[3], anti-neuronal[4], anti-ganglioside[5], anti-glial fibrillary acidic protein[6], anti-dsDNA, anti-N-methyl-d-aspartate (NMDA) receptors[7], anti-triose-phosphateisomerase[8], anti-SSA/Ro (Anti-Sjögren’s-syndrome-related antigen A, or anti-Ro)[9], anti-ribosomal P protein[10], anti-cardiolipin[11], and anti-alpha-internexin autoantibodies[12]. However, most of these autoantibodies can also be detected in other autoimmune diseases; only a small fraction of them (such as anti-GluRɛ2 and anti-NMDA)are specifically associated with neuropsychiatric disorders[3, 7]. Based on the current literature, we hypothesized that there are additional autoantibodies in CSF that can be used to diagnose or predict NPSLE specifically.

In a normal adult the CSF volume is only 125–150 mL, and the amount of sample that can be collected from one person is very limited. In addition, the concentration of immunoglobulin G (IgG) in CSF is~42 ± 21mg/L[13], much less than that of serum (1118 ± 251g/L)[14]. Therefore, it is more difficult to screen autoantibodies in CSF than in serum. Unfortunately, the autoantibodies in serum can not represent the autoantibodiesin CSF of NPSLE patients [7]. Traditional technologies such as phage display technology and western blot are not suitable for screening autoantibodies in individual CSF samples, because they also require more sample volume than patients can offer.

During the past decade, functional protein microarrays have become a powerful proteomics tool enabling novel discoveries in many biological fields[15–19].Within a functional protein microarray, thousands of individually purified proteins are immobilized on a solid phase. This allows parallel high-throughput detection of autoantibodies in a single experiment[20]with only trace amounts of samples. For example, protein microarrays have been used to profile the autoantigens in sera of patients with autoimmune hepatitis[21], primary biliary cirrhosis[22],SLE[23], and chronic kidney disease[24]. In addition, Roche and colleagues[25]applied high-density protein microarrays to profile autoantibodies in CSF, and anti-RBPJ (recombination signal binding protein for immunoglobulin kappa J region) autoantibodies were found in the CSF of patients with multiple sclerosis[26].

In the present study, we used a human proteome microarray to profile disease-related autoantibodies in the CSF of SLE patients with neuropsychiatric syndromes.

## Patients and Methods

The study was approved by the Ethics Committee of Peking Union Medical College Hospital. All patients provided written consent.

### Patients and Samples

Inpatients and outpatients of Peking Union Medical College Hospital during 2004–2011 were enrolled. SLE patients received diagnoses based on the criteria established by the American College of Rheumatology (ACR) in 1982,which was revised in 1997[27]; NPSLE patients fulfilled the ACR 1999 definition[2].A CSF specimen and matched serum specimen were collected on the same day from each patient. All samples were stored at—80°C.

Two cohorts of samples were used in the study. The first cohort was used to screen candidate autoantigens with the human proteome microarray, including CSF specimens of 12 NPSLE, 7 non-NPSLE, and 10 non-SLE(5 meningitis and 5 scoliosis) patients. The second cohort was for validation with a focused autoantigen microarray, including CSF specimens of 51 non-SLE(10 meningitis, 12 acute lymphocytic leukemia, 12 glioma,17 brain trauma), 45 NPSLE, and 28 non-NPSLE patients, and 41 matched serum specimens of 5 non-SLE(2meningitis, 2 acute lymphocytic leukemia, 1 glioma), 26 NPSLE, and 10 non-NPSLE patients.

### Quality assessment of human proteome microarray

The human proteome microarrays contained about 17000 unique human full-length proteins with an N-terminal GST (glutathione S-transferase) tag and some protein controls in duplicates[28]. These proteins were expressed in yeast and affinity-purified. Anti-GST monoclonal antibody (mAb) was used to assess the quality of the microarrays before the assay. Signal intensity from duplicate spots of human protein fit a unary linear regression model; R^2^ was calculated to evaluate the consistency of duplicate spots.

### Capture and visualization of antibodies on human proteome microarray

The procedures for the anti-GST and specimen assay with the human proteome microarray were performed as described previously[22], with minor modifications. Briefly, after blocking and incubation with 1:20 diluted CSF, 1:2000 diluted Cy5-labeled goat anti-human IgG antibody was applied to detect bound human IgG. Arrays were scanned with aLuxScan-10K/A scanner (CapitalBio, Beijing, China) using optimal settings (power = 95, photomultiplier tube = 850) in a 635-nm channel.

### Human proteome microarray data analysis

GenePix 5.1 was used to obtain microarray signal intensity. Fmedian/Bmedian was used to execute background correction and to normalize the intra-array signal intensity. *I*_*j*_denoted the normalized intensity of spot *j* on the protein microarray. All *I*_*j*_(control spots omitted) on each protein microarray were used to construct an intensity distribution curve. In general, if there is no positive hit on the protein microarray, the intensity distribution curve for the protein microarray is bilaterally symmetrical with the line *x* = 1 as the axis of symmetry. Here, positive hits resulted in an intensity distribution with a positively skewed distribution. Therefore, we inverted (mirrored) the actual intensity distribution curve on the left of line *x* = 1 to the right side, to fabricate a bilaterally symmetrical curve, and calculated the standard deviation (SD) for this new curve. We used mean_*i*_ to denote the average of *I*_*j*_(control spots omitted) on protein microarray_*i*_. The cutoff of the mean_*i*_+6·SD was applied to determine a positive hit on protein microarray_*i*_. Human proteins and various controls were considered as a positive feature only when both of their duplicate spots were simultaneously judged as a positive hit.

### Construction of focused autoantigen microarray

Twelve candidate autoantigens resulted from screening with the human proteome microarray, and 8autoantigen control proteins were used to construct a focused autoantigen microarray. Plasmid pEGH-bearing coding sequences of autoantigens were transformed into yeast strain Y258 and the proteins were expressed as GST-fusion proteins and purified by affinity chromatography. Spotting buffer, bovine serum albumin (BSA), GST, H5N1, human IgG, and mouse IgG were used as controls on the focused autoantigen microarray. GST protein was expressed from an empty pEGH vector.

**Fig 1:**
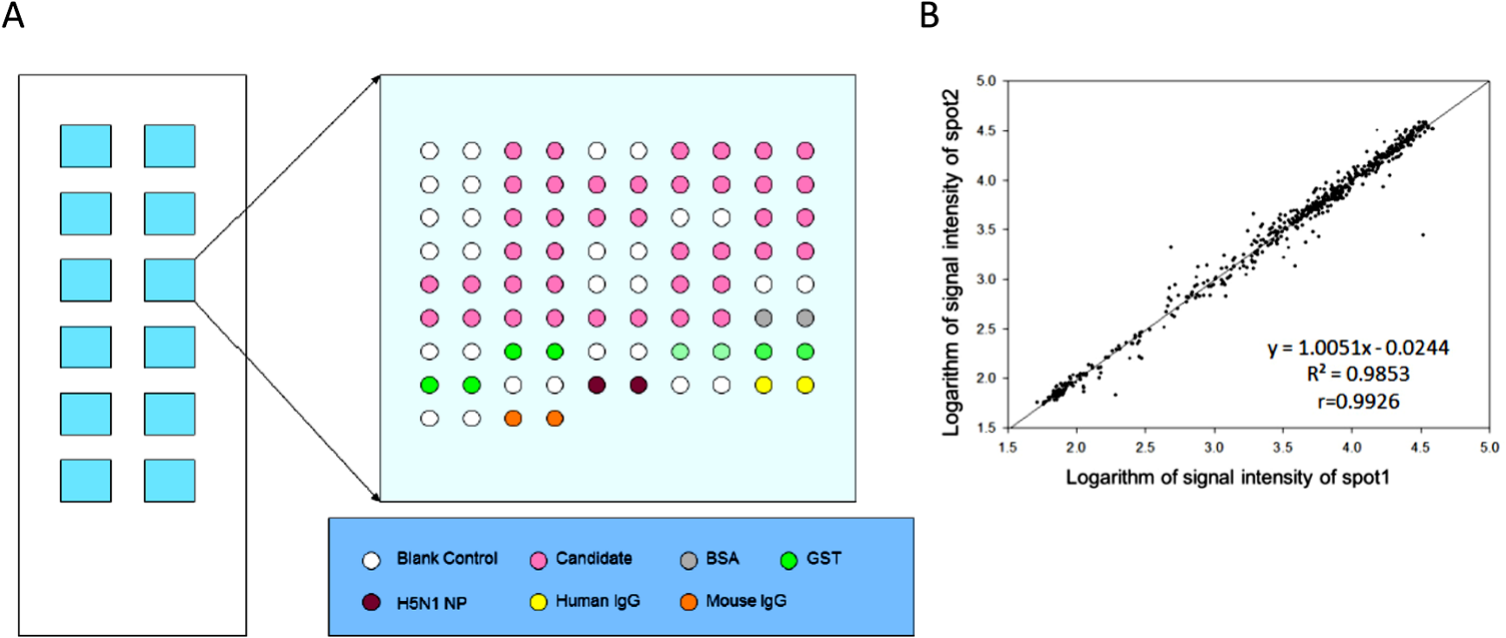
Focused autoantigen microarray. (A). Layout of the focused autoantigen microarray. The microarray slide contains 12 identical blocks. Each printed 20 autoantigens and various controls in duplicate. The shade of green denotes the concentration of GST protein. (B). Scatter plot of signal intensity of duplicate spots assayed with anti-GST antibody. Linear regression analysis was applied to quantify the consistency of signal intensity of duplicate spots.

BSA was purchased from Sigma-Aldrich. H5N1 protein, mouse IgG, and human IgG were supplied by Beijing Protein Innovation (Beijing). The Bradford method was applied to determine the concentration of proteins. The layout of the focused autoantigen microarray is displayed in Fig 1A. Proteins and controls were spotted on OPEpoxy glass slides (CapitalBio, Beijing, China) using a Perkin Elmerarray spotter (Perkin Elmer, MA, USA). Twelve identical blocks were aligned on a single slide, and every autoantigen or control had duplicate spots. The prepared microarrays were stored in vacuumed packages at 4°Cuntil assayed.

### Capture and visualization of antibodies on focused autoantigen microarray

One hundred and twenty-four CSF specimens (1:20 diluted) and 41 matched serum specimens (1:500 diluted) were assayed using the focused autoantigen microarray. Anti-GST mAb (1:2000) was used to measure the quality of the customized microarray. A SmartGrid (CapitalBio, Beijing, China) was fixed to the slide surface to form 12 identical chambers, and each chamber was blocked with3% BSA in PBST at 37°C for 1h. CSF or serum was diluted using3% BSA in phosphate-buffered saline with Tween 20 (PBST). Each diluted specimen was randomly pipetted into 2 different chambers and incubated at 37°Cfor 1h. Specimens were removed by centrifuging for 2 min at 2000 rpm in a 50-mL centrifuge tube, and PBST, preheated to 37°C,was used to wash the slide 3times for 10 min each.

After removing the PBST, Cy5-conjugated anti-human IgG or anti-mouse IgG was used to probe the primary antibodies bound to the slide. Secondary antibodies and the SmartGrid were removed from the slide. The slide was consecutively washed with preheated PBST and Milli-Q water 3 times for 10 min each. After removing the Milli-Q water and drying, the slides were scanned using theLuxScan-10K/A scanner at optimal settings (power = 95, photomultiplier tube = 850) for Cy5.

### Focused autoantigen microarray data analysis

The signal intensity of the spots on the microarray was extracted using Genepix 5.1. Because the autoantigens on the microarray were GST-fused proteins, the signals measured may come partially from the GST-tag, or other non-target-coding parts of the fusion proteins. To eliminate these potential interferences, we developed the following method to differentiate the positive feature on the focused autoantigen microarray.

GST proteins, expressed and purified from the empty pEGH vector, were spotted onto each block at 4concentrations. In each block, the signal intensity and natural logarithm of the molecular amount of GST proteins from the 4concentrations fit a unary linear regression equation. We assumed that proteins with the same molecular amount would have the same interference signal intensity. With this assumption, the interference signal intensity for a given autoantigen, denoted *I*_*p*_, could be deduced from the fitted unary linear regression equation by the molecular amount of autoantigen.The average mean intensity of the foreground pixels from duplicate spots for each autoantigen or control was characterized as the actual intensity (*I*_*a*_). For each autoantigen or control, we obtained a ratio for *I*_*a*_-to-*I*_*p*_. In CSF, we considered an autoantigen or control with a ratio ≥2, simultaneously in 2different assays, as positive spots. In the serum group, the ratio was set at ≥3.

### Auto-antigenicity validation by western blot

Five autoantigens were expressed: RPLP0, RPLP1, RPLP2, SS-A, and PCNA. The GST-tag was removed from the GST-fusion protein as previously described[29]. The recovered GST-free proteins (400ng) were resolved via 12% SDS-PAGE, and then transferred onto a polyvinylidene fluoride (PVDF) membrane (Φ = 0.45μm). Autoantibodies in diluted CSF (1:10) and serum (1:200) were detected by western blot[30].

### Statistical analysis

Fisher’s exact test, the chi-squared test, the Kruskal-Wallis test, and Spearman’s rank correlation coefficient analysis were used in our study using the R software package. *P*<0.05 was considered statistically significant.

## Results

### Screening CSF specimens using human proteome microarray

The human proteome microarrays held ~17000 individually purified full-length human proteins as GST-fusions. Since the reproducibility of protein microarray data is crucial for biomarker identification, we first assessed their quality by probing a human proteome microarray, randomly selected from the same printing batch, with anti-GST mAb. Linear regression analysis was applied to quantify the consistency of signal intensity of duplicate spots. A high correlation coefficient R^2^ of 0.98 was obtained, indicating that this batch of microarrays were of high quality (S1 Fig).

**Fig 2:**
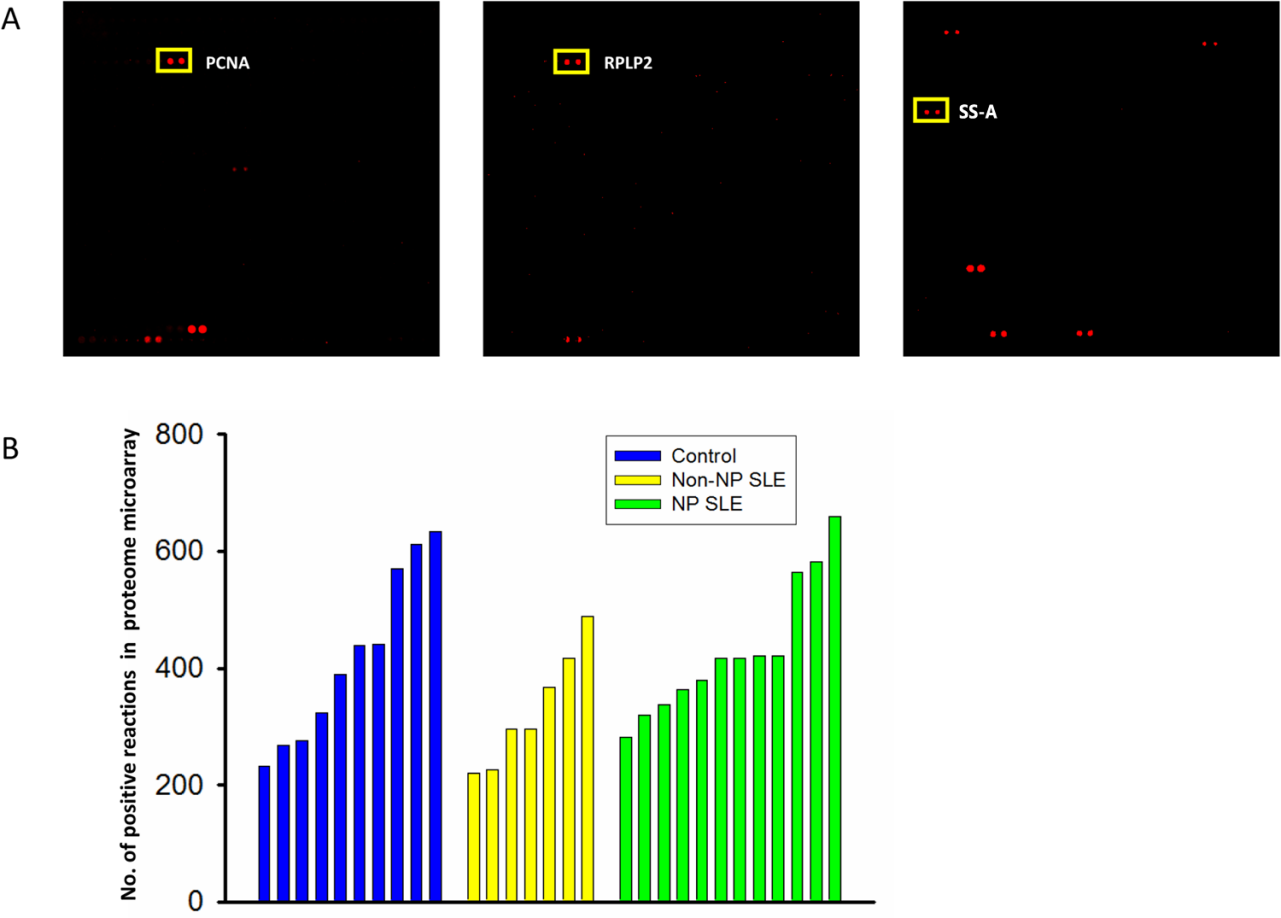
Detecting CSF autoantigens with the proteome microarray. (A). Representative scan images of a microarray, with target autoantigens in rectangles. (B). The number of positive reactions detected in three groups.

To identify NPSLE-specific CSF autoantibodies, we profiled the autoimmune reactivity by incubating the human proteome microarrays with 29 individual CSF samples from 12 NPSLE, 7 non-NPSLE, and 10 non-SLE patients, and then performed signal detection with Cy5-labeled anti-human IgG antibodies. Each of the 29 CSF specimens was incubated on a randomly selected human proteome microarray of the same printing batch. Human proteins that showed strong autoimmunogenicity could be readily detected in the CSF specimens (Fig 2A).Using a previously established protocol, protein spots showing positive signals on each individual protein microarray were identified, and the number of the positive features on each protein microarray was counted and displayed in a bar chart (Fig 2B). The Kruskal-Wallis test was used to compare the number of positive reactions among the NPSLE (431 ± 109), non-NPSLE (330 ± 92), and non-SLE control (419 ± 140) groups. However, the number of positive spots among the 3 groups were statistically similar (*P*< 0.05).

**Fig 3:**
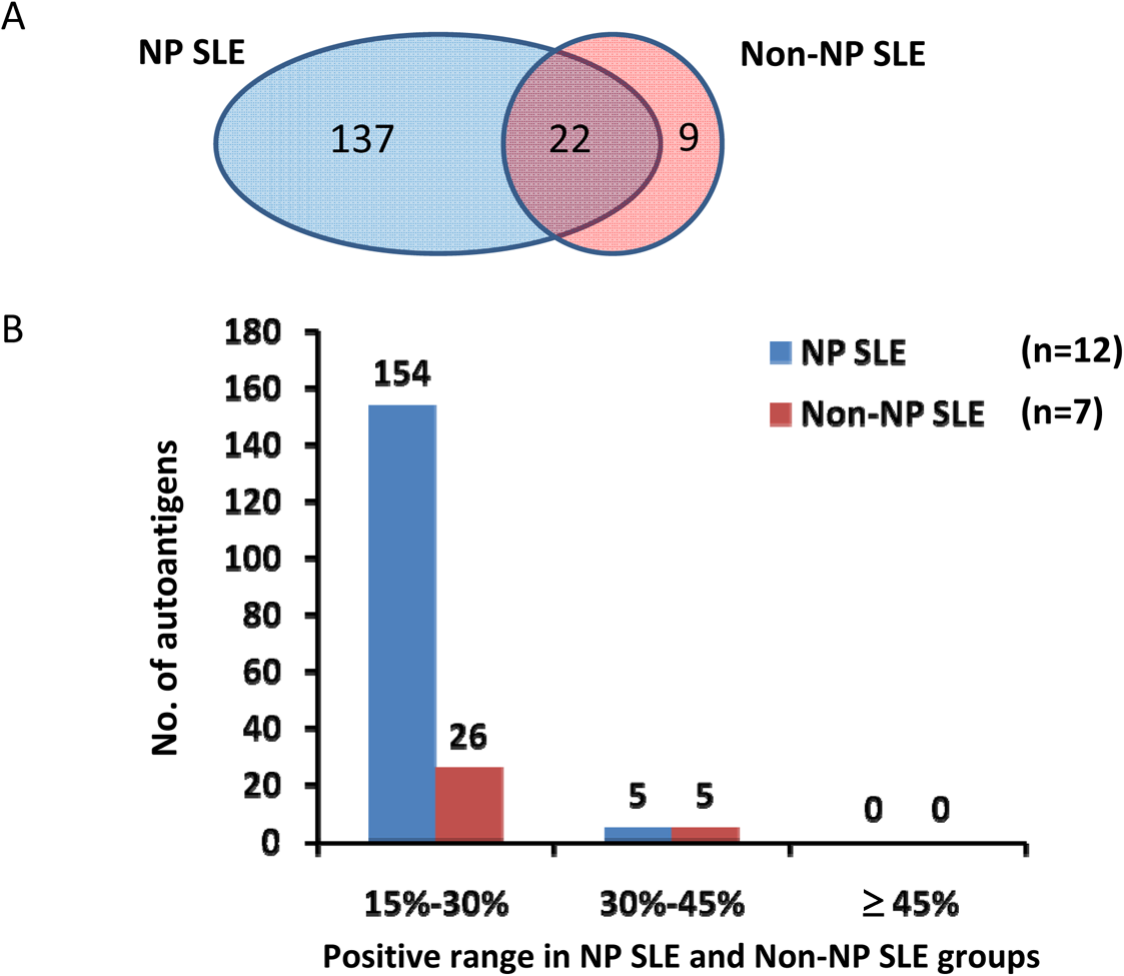
Autoantigens found in the NPSLE and non-NPSLE groups. (A). Venn Diagram of autoantigen distribution. (B). Distribution of autoantigens in various positive ranges /

To analyze the autoantibodies specifically present in the CSF of SLE patients, especially in the NPSLE patients, we first excluded the autoantigens that showed positive signals observed in the non-SLE control samples. By using a positive rate of 15% (ratio≥2) within the group as a cutoff, we found 159 and 31 autoantigens associated with the NPSLE and non-NPSLE patients, respectively; 22 were shared(Fig 3A). The associations between the number of autoantigens and the positive range in NPSLE and non-NPSLE groups are summarized in Fig 3B. As the positive rate increased, the number of autoantigens decreased dramatically in both the NPSLE and non-NPSLE groups. In addition, we found few autoantibodies with high sensitivity in either the NPSLE or the non-NPSLE specimens.

### Network enrichment of autoantibodies from CSF of NPSLE or non-NPSLE patients

Ingenuity Pathway Analysis(IPA), a web-based functional analysis tool for comprehensive omic data, was used to identify potential biological function enrichment among those autoantigens associated with CSF obtained from NPSLE and non-NPSLE patients (S1–S4 Tables). IPA gives a network score, indicating the degree of relevance of molecules of interest that interact with other molecules in the IPA’s database (the “Ingenuity Knowledge Base”). The score is based on the hypergeometric distribution and is calculated as the negative log of the *P*-value, using the right-tailed Fisher’s exact test. The 137 autoantigens specifically associated only with NPSLE specimens were found significantly enriched in neurological disease (with a score of 43). Autoantigens common in NPSLE and non-NPSLE were prone to participate in an inflammatory response (with a score of 29).

### Validation of autoantigens in extended CSF specimens using the focused autoantigen microarray

One major concern with biomarker identification is statistical overfitting, caused by a relatively small cohort size or complex factors or variables involved with a particular disease. To minimize overfitting, we recruited a much larger cohort of CSF specimens to validate those autoantigen candidates identified on the human proteome microarrays. Using our previously established two-phase approach[21], we fabricated a focused autoantigen microarray (Fig 1A) with selected candidate proteins associated with NPSLE. Fifteen human proteins were selected, because they appeared in ≥30% of NPSLE patients with >90% specificity relative to the controls (Table 1).Given that anti-Sm, anti-rRNP, and anti-PCNA autoantibodies were frequently detected in the serum of SLE patients, the autoantigens recognized by these autoantibodies, including SNRPB (small nuclear ribonucleoprotein polypeptides B and B1), SNRPD1 (small nuclear ribonucleoprotein D1 polypeptide), RPLP0, RPLP1, RPLP2, and PCNA, were also included in the focused autoantigen microarray. Protein EIF2C1 was also included as an independent control.

**Table 1:** List of candidate NPSLE autoantigens identified by proteome microarray.

Autoantigen	NPSLE ^a^	Non-NPSLE^b^	Control^c^
SS-A	41.67%(5)	42.86% (3)	0.00% (0)
MAD1L1	41.67% (5)	42.86% (3)	0.00% (0)
CSRP3	41.67% (5)	28.57% (2)	10.00% (1)
C16orf73	33.33% (4)	0.00% (0) ^d^	0.00% (0)
ATRX	33.33% (4)	14.29% (1)	0.00% (0)
C9orf11	33.33% (4)	28.57% (2)	0.00% (0)
C1orf212	33.33% (4)	0.00% (0)	0.00% (0)
APTX	33.33% (4)	14.29% (1)	10.00% (1)
DLC1	33.33% (4)	14.29% (1)^d^	10.00% (1)
INDO	33.33% (4)	14.29% (1)	10.00% (1)
H3	33.33% (4)	28.57% (2)^d^	10.00% (1)
CHRND	33.33% (4)	14.29% (1)	10.00% (1)
RANGNRF	33.33% (4)	14.29% (1)	10.00% (1)
VPS29	33.33% (4)	42.86% (3)	10.00% (1)
CENPM	33.33% (4)	0.00% (0)	10.00% (1)

^a^n = 12;

^b^n = 7;

^c^n = 10;

^d^Three of the listed autoantigens were not included in the focused autoantigen microarray.

As described previously, purified candidate autoantigen proteins, as well as positive and negative controls, were spotted in duplicate in a 2×6 format to form 12 identical blocks on each glass slide[21]. Each CSF specimen was incubated with 2blocks printed on 2 separate slides, and only when a candidate protein was recognized as positive in the 2 duplicated assays by the same CSF specimen, was it considered to be a true positive.CSF specimens (in total, 124) collected from 51 non-SLE, 45 NPSLE, and 28 non-NPSLE subjects were assayed on the focused microarrays.

The consistency of discriminations in2 independent assays reached 95.8% among all CSF specimens tested. The positive rates of autoantibodies against RPLP0, RPLP1, RPLP2, and SS-A were significantly higher in the NPSLE group than in then on-NPSLE or non-SLE groups(Table 2). Furthermore, anti-SS-A autoantibodies in CSF specimens showed a significantly higher positive rate in the NPSLE group than in the non-NPSLE group (*P* = 0.045). All the autoantigens are listed according to the positive rates, in decreasing order, in the NPSLE group.

**Table 2:** Positive rate of autoantibodies observed in CSF validation cohort with focused autoantigen microarrays.

Autoantigen	Positive rate of corresponding autoantibodies			*P* value ^a^		
	Control^b^	Non-NPSLE^c^	NPSLE^d^	Non-NPSLE cf. control	NPSLE cf.control	Non-NPSLE cf. NPSLE
RPLP2	0.00%	25.00%	33.33%	0.0004	<0.0001	0.4505
SS-A	0.00%	10.71%	31.11%	0.0414	<0.0001	0.0450
RPLP1	0.00%	25.00%	28.89%	0.0004	<0.0001	0.7172
RPLP0	0.00%	21.43%	26.67%	0.0014	<0.0001	0.6136
CHRND	5.88%	7.14%	4.44%	1	1	0.6350
PCNA	0.00%	3.57%	4.44%	0.3544	0.2171	1
EIF2C1	0.00%	3.57%	2.22%	0.3544	0.4688	1
SNRPB^d^	0.00%	0.00%	2.22%	1	0.4688	1
SNRPB(c) ^d^	0.00%	0.00%	2.22%	1	0.4688	1
C9orf11	1.96%	0.00%	2.22%	1	1.	1
ATRX	0.00%	0.00%	0.00%	1	1	1
CENPM	0.00%	0.00%	0.00%	1	1.	1
CSRPS	0.00%	0.00%	0.00%	1	1	1
SNRPD1	0.00%	7.14%	0.00%	0.1227	1	0.1438
VPS29	0.00%	0.00%	0.00%	1	1	1
MADIL1	0.00%	0.00%	0.00%	1	1.	1
APTX	0.00%	0.00%	0.00%	1	1	1
ID01	0.00%	0.00%	0.00%	1	1	1
C1orf212	0.00%	0.00%	0.00%	1	1	1
RANGRF	0.00%	0.00%	0.00%	1	1	1

^a^ Chi-squared test or Fisher’s exact test were used to compare the positive rates between 2 groups;

^b^n = 51;

^c^n = 28;

^d^n = 45, autoantigens were listed according to the positive rates at decreasing order;

^e^SNRPB had 2 copies in the same block to be used as a quality evaluation index.

### Association of anti-SS-A autoantibodies with NPSLE

Since the prevalence of anti-SS-A in CSF specimens was significantly different between the NPSLE and non-NPSLE groups, we wanted to determine further whether it had any association with particular neuropsychiatric syndromes. Seventy-three CSF specimens from SLE patients were divided into anti-SS-A^+^ and anti-SS-A^—^groups. In each group, neuropsychiatric syndromes were recorded and *P*-values were calculated using Fisher’s exact test or the chi-squared test (Table 3). We found that anti-SS-A autoantibodies were significantly associated with neurological syndromes of the central nervous system (*P* = 0.009).

**Table 3:** Association of anti-SS-A with neuropsychiatric syndromes of SLE.

Neuropsychiatric syndromes	Anti-SS-A (+) ^a^	Anti-SS-A (-)^b^	*P* value^c^
Central nervous system	82.35%	46.43%	0.0091
Aseptic meningitis	0.00%	0.00%	1
Cerebrovascular disease	5.88%	5.36%	1
Demyelinating syndrome	0.00%	0.00%	1
Headache	41.18%	26.79%	0.3654
Chorea	0.00%	1.79%	1
Myelopathy	0.00%	3.57%	1
Seizure disorders	23.53%	8.93%	0.1990
Acute confusional state	11.76%	10.71%	1
Anxiety disorder	5.88%	3.57%	0.5543
Cognitive dysfunction	29.41%	14.29%	0.1656
Psychosis	35.29%	19.64%	0.2017
Peripheral nervous system	17.65%	10.71%	0.4264
Guillain-Barré syndrome	0.00%	0.00%	1
Autonomic disorder	0.00%	0.00%	1
Mononeuropathy^d^	5.88%	3.57%	0.5543
Myasthenia gravis	0.00%	0.00%	1
Neuropathy,cranial	11.76%	7.14%	0.6185
Plexopathy	0.00%	0.00%	1
Polyneuropathy	0.00%	1.79%	1

^a^n = 17;

^b^n = 56;

^c^ chi-squared test or Fisher’s exact test were used to compare the positive frequencies between 2 groups;

^d^ single or multiplex.

### Correlation of autoantibody titers in matched CSF and serum specimens

**Fig 4:**
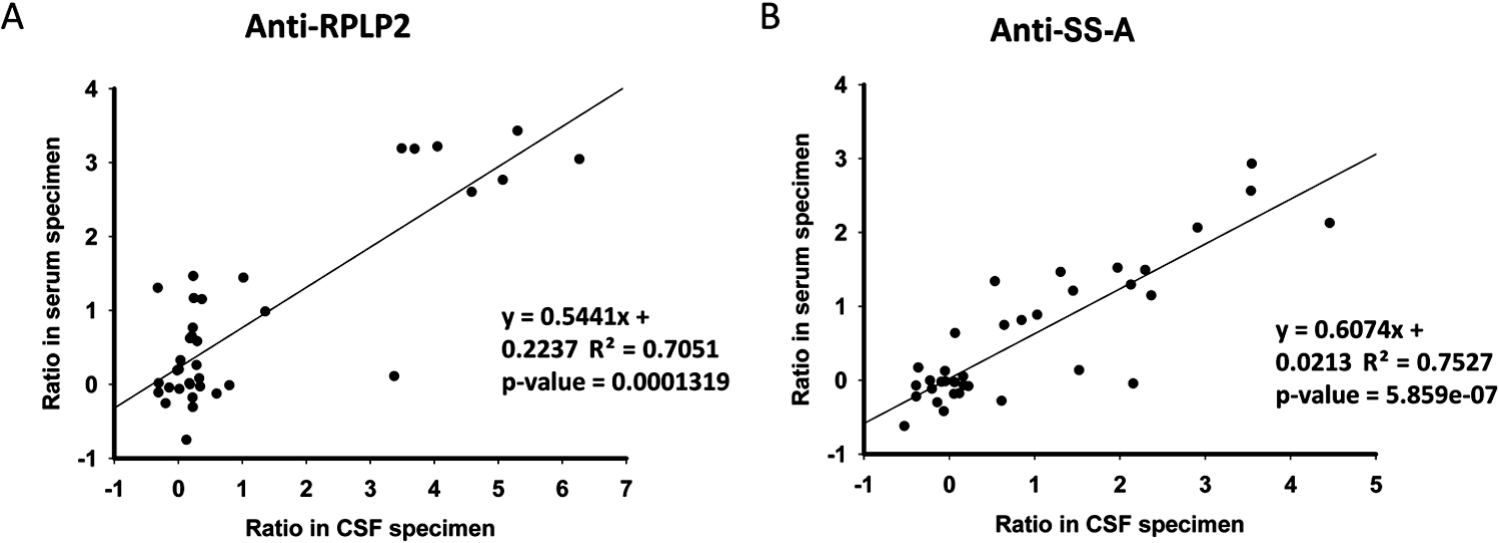
Titers of 2 autoantibodies in matched CSF and serum specimens. Scatter plots of titers of anti-RPLP2 (A) and anti-SS-A (B) in matched CSF and serum. Ratio in CSF specimen is the normalized signal.

Although autoantibodies were previously reported detectable in both CSF and serum samples in SLE patients, a potential correlation between CSF- and serum-based autoantibodies has not been extensively investigated[31]. Therefore, we decided to profile the autoimmune activities of 41 CSF-matched serum samples, collected from 5non-SLE, 26 NPSLE, and 10 non-NPSLE subjects, and compare the autoantibody titers between the matched CSF and serum samples by Z-score (Fig 4).We focused on anti-RPLP2 and anti-SS-A autoantibodies, because they had a relatively higher prevalence in CSF specimens of SLE patients. As the concentration of autoantibodies in serum is generally higher than that in CSF, 1:500 and 1:20 dilutions were used for the serum and CSF samples, respectively. To ensure accurate comparison, we optimized the scanning condition to avoid any saturated signals of those positive spots.

The Z-scores between the CSF and serum samples showed rather high correlations for both autoantigens, with correlation coefficients of 0.71 and 0.75 for RPLP2 and SS-A, respectively. Statistical analyses also revealed significant *P*-values between the CSF and serum samples (1.32E–04 for RPLP2; 5.86E–07 for SS-A).Interestingly, the correlation coefficient should be even higher for those matched samples with Z-scores>2 (positive signals). Therefore, autoantibody titers of the CSF and serum samples strongly correlated, indicating that autoantibodies found in the CSF were probably due to leakage through the compromised blood-brain barriers from the blood.

### Five autoantibodies in serum and CSF specimens validated by immunoblot analyses

Because of the limited availability of the CSF specimens, we only employed 15 CSF specimens in the validation assay using the standard immunoblotting assays. Previously, anti-RPLP0, anti-RPLP1, and anti-RPLP2 autoantibodies had been widely reported in CSF specimens of SLE patients[10, 32]. Therefore, we focused on validating the anti-TROVE2 and anti-PCNA autoantibodies in CSF specimens of SLE patients, with RPLP0, RPLP1, and RPLP2 as positive controls. SS-A and PCNA, as well as the 3RPLP proteins, were purified as GST fusion to perform immunoblot analysis with matched CSF and serum samples.

**Fig 5:**
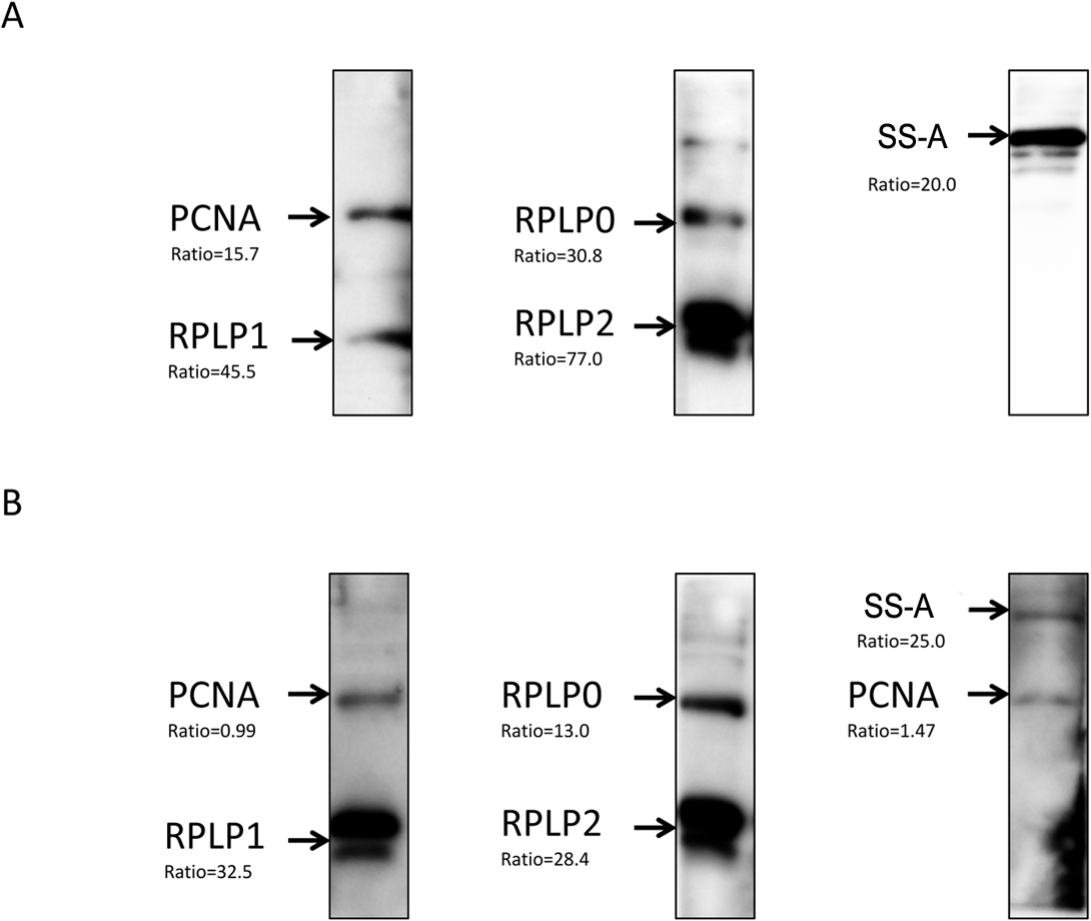
Immunoblot analyses of CSF and serum specimens validated the 5autoantigens. The purified recombinant proteins were resolved viaSDS-PAGE, transferred to a PVDF membrane, and subjected to western blot with (A) CSF and (B) serumspecimens. The names of the recombinant proteins are indicated on the left side of images and the ratios underneath resulted from the microarray experiment.

Autoantibodies against both SS-A and PCNA were readily detected in both CSF and serum samples (Fig 5).As expected, anti-RPLP0, anti-RPLP1, and anti-RPLP2 autoantibodies were also detected. In summary, 2 novel autoantigens, SS-A and PCNA, discovered through the human proteome microarray-based approach, were validated as biomarkers in CSF.

## Discussion

Under normal conditions, the integrity of the blood-brain barrier helps maintain the homeostasis of the central nervous system. When the integrity is undermined under pathological conditions, such as NPSLE, substances in the blood will leak into the central nervous system, resulting in changes in the properties and composition of the CSF. Based on this notion, laboratory results from CSF are often used to assist clinical diagnosis. Indeed, autoantibodies have been detected in the CSF of SLE patients; some of them are correlated with neuropsychiatric syndromes[3, 7, 32].

In this study, we chose to use a human proteome microarray, comprising17000 full-length individually purified human proteins as an unbiased platform, to survey for novel NPSLE-associated autoantibodies in the CSF of SLE patients. We found that the prevalence of disease-associated autoantibodies in the CSF of SLE patients was low (Fig 3B), <45% in both the NPSLE and non-NPSLE groups. Only a few autoantibodies showed a positive rate, in the range of 30% to 45%, and most of them varied from 15% to 30%. This result is consistent with most autoantibody studies reported in SLE, suggesting that multiplexed detection against a series of autoantibodies is more desirable to achieve higher sensitivity and specificity in NPSLE diagnosis.

Although the pathology of NPSLE and non-NPSLE is quite different, our unbiased survey on the human proteome microarray led to the identification of 22 shared autoantigens between these2 subtypes of SLE. Interestingly, bioinformatics analyses of these 22 autoantigens revealed a significant enrichment in the inflammatory response (score = 29). Because SLE is a prototype autoimmune disease, and because activation of autoreactive B or T cells leads to chronic tissue inflammation and often irreversible structural and functional damages[33], our analysis suggests a potential candidate list of autoantigens that might better explain the etiology of SLE. Presumably accompanied by increased inflammation in patients, the abnormal over expression of inflammatory molecules further triggers the production of new autoantibodies, which may be used as regulatory factors to control the abnormal inflammatory response in a negative feedback manner.

In NPSLE, autoantibodies directly cause damage to the nervous system. In adult mouse models, when the blood-brain barrier is compromised, anti-NMDA receptor autoantibodies can access the brain and elicit neuronal death with ensuing cognitive dysfunction and emotional disturbance[34]. Injection of anti-ribosomal-P protein (anti-P) autoantibodies into the brains of living rats triggered neuronal death by apoptosis[35]. Indeed, our informatics analysis via IPA of the 137 autoantigens that were identified only among NPSLE patients revealed a significant enrichment associated with neurological diseases (score = 43). It is reasonable to believe that some of them may be directly involved in the injury of the nervous system. However, because too many distinct neuropsychiatric syndromes are associated with NPSLE, it would require profiling a huge cohort of NPSLE patients (>1000) on the human proteome microarrays to tease out syndrome-specific autoantigens to better understand the underlying etiology. Due to the cost of the human proteome microarrays, this was impractical for our current studies.

Autoantibodies in CSF may come from intrathecal antibody synthesis[36] or leakage of serum autoantibodies due to impairment of the blood-brain barrier function[37]. Because anti-RPLP2 and anti-SS-A exhibited relatively higher positive rates in the focused autoantigen microarray experiment, we investigated the correlation of titers of autoantibodies in CSF and matched serum samples from the same patients. We found that the titers of anti-RPLP2 and anti-SS-A in matched CSF and serum specimens significantly correlated(*P*<0.01). This indicated that autoantibodies in the CSF of SLE patients were mainly from the leakage of autoantibodies through the compromised blood-brain barrier.

In this study, we detected and validated that anti-SS-A autoantibodies are significantly associated with NPSLE, based on both protein microarray and standard immunoblot assays. To our knowledge, this is the first report to firmly connect anti-SS-A autoantibodies with neuropsychiatric symptoms in SLE, although anti-SS-A was previously detected in the CSF and sera of NPSLE patients[9]. Importantly, the prevalence of anti-SS-A autoantibodies was significantly higher (*P* = 0.045) in the NPSLE group (31.11%) than in the non-NPSLE group (10.71%). Further analysis showed that anti-SS-A in CSF specimens was associated with neurological syndromes of the central nervous system in SLE patients (*P* = 0.009). SS-A is an RNA-binding protein that binds to misfolded non-coding RNAs, pre-5S rRNA, and several small cytoplasmic RNA molecules known as Y RNAs. It may stabilize some of these RNAs and protect them from degradation. It is not clear whether anti-SS-A antibodies participate in the etiology of neurological syndromes.

## Conclusions

Previously, anti-PCNA autoantibodies had only been reported in<5% of the serum samples of SLE patients[38]. In this study, for the first time, we detected anti-PCNA autoantibodies in the CSF of SLE patients, which was further validated by standard immunoblot assay. The correlation between autoantibodies targeting RPLP and NPSLE is controversial[39]. Our study found that anti-RPLP (P0, P1, P2) in CSF did not differ significantly between the NPSLE and non-NPSLE groups. We expect that by profiling larger NPSLE and related cohorts on human proteome microarrays, we will be able to identify more and probably better autoantigens that can further tease out syndrome-specific autoantibodies in NPSLE.

## Supporting Information

S1 Fig Quality measurement of proteome microarray. Randomly selected proteome microarray was hybridized with anti-GST mAb, and the scanning result is displayed in S1A Fig. Intensity of duplicate spots was extracted to draw scatter plot and calculate intensity consistency. Linear regression analysis was applied to quantify the consistency of signal intensity of duplicate spots in S1B Fig.
(TIF)

S1 Table Top network functions associated with 31 non-NPSLE autoantigens. (DOC)

S2 Table Top network functions associated with 137 NPSLE autoantigens. (DOC)

S3 Table Top network functions associated with 159 NPSLE autoantigens. (DOC)

S4 Table Top network functions associated with 22 common autoantigens. (DOC)

S5 Table CSF on human proteome microarray. (XLS)
