# Supplementary figures and images for: Autoantibody Profiling on Human Proteome Microarray for Biomarker Discovery in Cerebrospinal Fluid and Sera of Neuropsychiatric Lupus
Source: PLoS One. 2015 May 8;10(5):e0126643. doi: 10.1371/journal.pone.0126643 (PMC4425696; doi:10.1371/journal.pone.0126643)

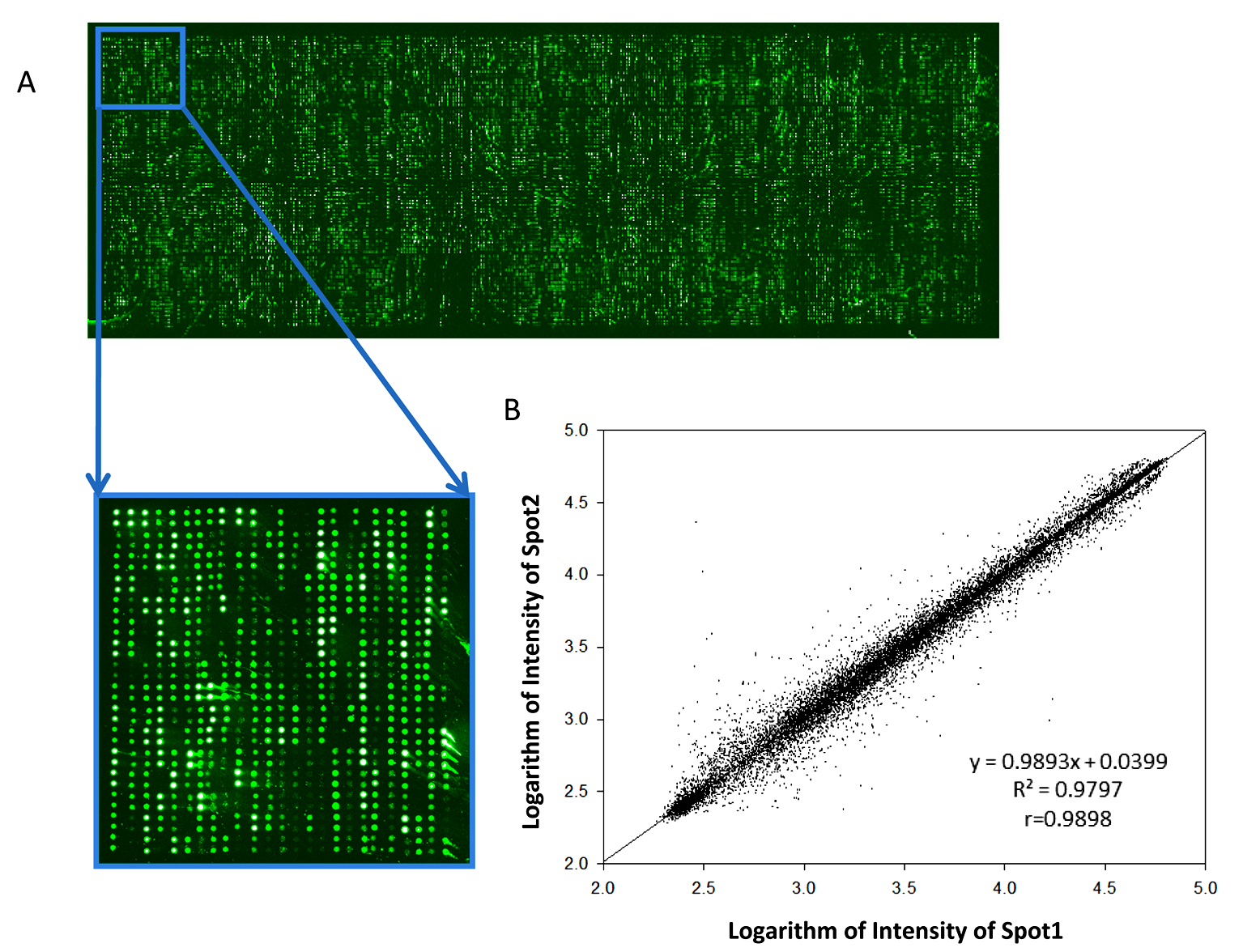

Supplement: S1 Fig — Randomly selected proteome microarray was hybridized with anti-GST mAb, and the scanning result is displayed in S1A Fig. Intensity of duplicate spots was extracted to draw scatter plot and calculate intensity consistency. Linear regression analysis was applied to quantify the consistency of signal intensity of duplicate spots in S1B Fig. (TIF) [file pone.0126643.s001.tif]
