# Supplementary material for: Autoantibody Profiling on Human Proteome Microarray for Biomarker Discovery in Cerebrospinal Fluid and Sera of Neuropsychiatric Lupus
Source: PLoS One. 2015 May 8;10(5):e0126643. doi: 10.1371/journal.pone.0126643 (PMC4425696; doi:10.1371/journal.pone.0126643)
Supplement: S1 Table — (DOC) [file pone.0126643.s002.doc]

**S1** Table Top network functions associated with 31 Non-NPSLE autoantigens

| **ID** | **Associated Network Functions** | **Score*** |
| --- | --- | --- |
| **1** | Amino Acid Metabolism, Small Molecule Biochemistry, Inflammatory Disease | 23 |
| **2** | Cellular Function and Maintenance, DNA Replicaiton, Recombination, and Repair, Organ Morphology | 13 |
| **3** | Cellular Assembly and Organization, Cellular Function and Maintenance, DNA Replication, Recombination, and Repair | 2 |
| **4** | Drug Metabolism, Protein Synthesis, Cardiovascular Disease | 2 |
| **5** | Cancer, Gastrointestinal Disease, Cellular Assembly and Organization | 2 |

*The network score indicates the degree of relevance to the Network Eligible Molecules in the dataset and is the negative log of this p-value calculated with the right-tailed Fisher’s Exact Test.
