# Supplementary material for: Autoantibody Profiling on Human Proteome Microarray for Biomarker Discovery in Cerebrospinal Fluid and Sera of Neuropsychiatric Lupus
Source: PLoS One. 2015 May 8;10(5):e0126643. doi: 10.1371/journal.pone.0126643 (PMC4425696; doi:10.1371/journal.pone.0126643)
Supplement: S2 Table — (DOC) [file pone.0126643.s003.doc]

**S2** Table Top network functions associated with 137 NPSLE autoantigens

| **ID** | **Associated Network Functions** | **Score** |
| --- | --- | --- |
| **1** | Cancer, Neurological Disease, Cell Cycle | 43 |
| **2** | Gene Expression, Cell Cycle, Cell Morphology | 18 |
| **3** | Cellular Development, Hair and Skin Development and Function, Cell Death | 16 |
| **4** | Cellular Movement, Hematological System Development and Function, Immune Cell Trafficking | 16 |
| **5** | Cellular Development, Cellular Growth and Proliferation, Hematological System Development and Function | 16 |

*Twenty-two autoantibodies common in Non-NPSLE have been removed, only 137 autoantibodies were involved in this IPA analysis
