# Supplementary material for: Autoantibody Profiling on Human Proteome Microarray for Biomarker Discovery in Cerebrospinal Fluid and Sera of Neuropsychiatric Lupus
Source: PLoS One. 2015 May 8;10(5):e0126643. doi: 10.1371/journal.pone.0126643 (PMC4425696; doi:10.1371/journal.pone.0126643)
Supplement: S3 Table — (DOC) [file pone.0126643.s004.doc]

**S3 Table** Top network functions associated with 159 NPSLE autoantigens

| **ID** | **Associated Network Functions** | **Score** |
| --- | --- | --- |
| **1** | Cancer, Dermatological Diseases and Conditions, Cell Cycle | 49 |
| **2** | Inflammatory Response, Cellular Assembly and Organization, Cellular Function and Maintenance | 23 |
| **3** | Gastrointestinal Disease, Organismal Injury and Abnormalities, Cell Cycle | 21 |
| **4** | Cell-mediated Immune Response, Cellular Development, Cellular Function and Maintenance | 21 |
| **5** | Gastrointestinal Disease, Hepatic System Disease, Infectious Disease | 17 |
