# Supplementary material for: Autoantibody Profiling on Human Proteome Microarray for Biomarker Discovery in Cerebrospinal Fluid and Sera of Neuropsychiatric Lupus
Source: PLoS One. 2015 May 8;10(5):e0126643. doi: 10.1371/journal.pone.0126643 (PMC4425696; doi:10.1371/journal.pone.0126643)
Supplement: S4 Table — (DOC) [file pone.0126643.s005.doc]

**S4 Table** Top network functions associated with 22 common autoantigens

| **ID** | **Associated Network Functions** | **Score** |
| --- | --- | --- |
| **1** | Inflammatory Disease, Inflammatory Response, Renal Inflammation | 29 |
| **2** | Cellular Assembly and Organization, Cellular Function and Maintenance, DNA Replication, Recombination, and Repair | 3 |
| **3** | Drug Metabolism, Protein Synthesis, Cardiovascular Disease | 3 |
| **4** | Embryonic Development, Tissue Morphology, Nervous System Development and Function | 2 |
